# The Development of a Virtual World Problem-Based Learning Tutorial and Comparison With Interactive Text-Based Tutorials

**DOI:** 10.3389/fdgth.2021.611813

**Published:** 2021-04-20

**Authors:** Trupti Jivram, Sheetal Kavia, Ella Poulton, Aurora Sésé Hernandez, Luke A. Woodham, Terry Poulton

**Affiliations:** e-Learning Unit, Centre for Technology in Education, St George’s University of London, London, United Kingdom

**Keywords:** interactive tutorials, D-PBL medicine, virtual patients, problem-based learning, virtual worlds

## Abstract

Collaborative learning through case-based or problem-based learning (PBL) scenarios is an excellent way to acquire and develop workplace knowledge associated with specific competencies. At St George's, University of London we developed an interactive online form of decision-based PBL (D-PBL) for our undergraduate medical course using web-based virtual patients (VPs). This method of delivery allowed students to consider options for clinical management, to take decisions and to explore the consequences of their chosen actions. Students had identified this as a more engaging type of learning activity compared to conventional paper-based/linear PBL and demonstrated improved exam performance in controlled trials. We explored the use of Second Life (SL), a virtual world and immersive 3D environment, as a tool to provide greater realism than our interactive image and text-based D-PBL patient cases. Eighteen separate tutorial groups were provided with their own experience of the same patient scenario in separate locations within the virtual world. The study found that whilst a minority of students reported that the Second Life experience felt more realistic, most did not. Students favored the simpler interaction of the web-based VPs, which already provided them with the essential learning needed for practice. This was in part due to the time proximity to exams and the extra effort required to learn the virtual world interface. Nevertheless, this study points the way towards a scalable process for running separate PBL sessions in 3D environments.

## Introduction

For students in workplace or competency-led courses, collaborative learning through case-based or problem-based learning (PBL) scenarios is an excellent way to acquire and develop workplace knowledge associated with specific competencies, and diagnostic accuracy (1, 2).

The current pandemic has resulted in a swift and dramatic shift away from face-to-face small group learning, and as groups begin to engage more remotely, educators will search for tools which will help compensate for this loss of the more engaging physical presence. An obvious addition would be to add tools which challenge groups to solve tasks collaboratively online, rather than passively follow a fixed narrative. Our work in virtual worlds did not attempt to establish online collaboration, as that was not the primary need at that time. The collaborative space was still the face-to-face interaction based in a single room that is associated with traditional PBL, along with an in-person facilitator. Our goal was that the PBL was run as a blended activity mediated by the group collectively working with a single avatar in the virtual world to provide both the cues for discussion, and the available interactions and decisions at any given time.

Although PBL has proved effective and reasonably popular for more than 60 years, this traditional approach using “paper” cases has been limited by this medium of delivery, in two main ways.

First, traditional paper-based cases were linear, – which could only proceed in a single direction and did not allow students to take own decisions and explore the consequences of their actions. Consequently, at points of acute management, learners can only follow the path set out on the page. Such cases have limited use in developing clinical understanding, competency or reasoning, and are unrealistic for emulating real life, where there are frequently several ways to tackle a problem and mistakes made may not be immediately obvious. The unfolding “paper” case is never able to offer the learner the opportunity to take charge of the scenario or control the management of the patient, thus limiting the role of students as being solely observers.

Secondly, in the modern era, PBL development needs to explore the opportunity for more immersive and engaging methods of delivering the cases that utilize interactive visually oriented technologies. The need for this has been particularly reinforced during the COVID-19 pandemic in 2020, when much of medical education has moved online in an extremely short time frame.

The PBL cases at St George's differ from the normal linear narrative for a written PBL case in that each of the online narrative will contain a number of branching points which allow students to choose between a range of different actions or interpretations. Different branches have different consequences leading to different case outcomes; the students experience the consequences of good and bad decision, and must proceed from that point, just as would have to do in real life and underpinned by cognitive learning theories that focus on linearity problem-solving and the importance of attaining the right outcome (3). A PBL case delivered in a virtual world offers students safe practice, procedural experience, exposure to unseen conditions or diseases and above all, immersive decision-making opportunities (4, 5).

Several studies have considered the use of computer-aided instruction for variants of student-activated learning (6–8). In light of the experience of moving PBL online at St George's, University of London due to the pandemic, we were minded to reflect on our range of previous work toward developing PBL as a blended learning activity, in which we attempted to address the issues described by replacing linear paper cases with online interactive scenarios (9). We believed that it was critical for us to reflect upon this “journey” of iterative changes over more than a decade, such that we would be better able to identify positive improvements that can be made.

St George's has a Transitional year (T-year) within the medical course where the learning is based on PBL. This year is the cross-over period between campus-based learning and clinical apprenticeship, which brings together school-leaver, widening access and graduate-entry students with a total yearly intake of ~260 students. These students are organized into groups of 7–8, each in its individual location or “baseroom” to work through their PBL.

We had approached the first of the PBL limitations described by developing a more interactive online form of decision-based PBL (D-PBL) (10), which allows students to consider options for clinical management as the cases unfold, to take decisions and to explore the consequences of their chosen actions. Students had identified this as a more engaging learning activity than their conventional paper PBL, and in randomized trials, learners who experienced D-PBL exhibited improved exam performance (11).

Facilitator and student feedback identified that authentic decision making is a significant factor in student engagement (9). Based upon this idea, and building upon established principles for effective case design which establish the importance of providing multiple cues to stimulate discussion (12) we decided in 2010 to explore the use of a virtual world in our PBL tutorials to attempt to provide a more engaging, immersive environment and increase the range of available interactions to students at any given time, beyond the more limited range of menu-based choices available in other online formats. Previous studies had suggested a wide range of roles for virtual worlds in medical education: patient safety (13) skills training (8) social interaction for inter-professional training (14) and for postgraduate Continuing Medical Education (15).

Virtual worlds are three-dimensional online environments which users can adapt to their own needs. By graphically mimicking real-world situations, they can be used to tell a story, a story which the user can interact with and to some extent control its outcome. In this regard, it has similarities with our own D-PBL. The facilitation of teaching and learning through the use of technologies such as virtual worlds has been widely explored in higher education (16, 17). There have been many discussions about the uses and advantages of using virtual worlds (18, 19) but it is more relevant here to consider whether it could bring value to PBL in medical education.

Within an associated project, a trial was conducted for the replacement of Paramedic workplace practice teaching within an immersive 3D virtual world environment to explore whether it could provide greater realism and encourage active decision-making. Virtual patient (VP) scenarios were designed within the virtual world Second Life (SL) (20) by Linden Labs (21) to be used by learners working in groups of four remotely to each other. Feedback established that, despite some technology barriers, SL had the potential to provide a more authentic learner environment than paper or web-based PBL (22). The case study suggested that virtual worlds could offer greater realism, whilst retaining the active collaborative decision-making element of D-PBL. We therefore elected to develop a PBL tutorial that used virtual worlds as an alternative to our interactive online D-PBL, while still retaining the blended setting with students collaborating in a physical room.

Our first research question for a PBL in a virtual world setting was, could an immersive 3D environment provide improved realism over interactive image and text based PBL patient cases, and would that immersion lead to greater student engagement, making the decisions taken more memorable?

Our second research question for PBL was a practical one; was it possible to give each group the opportunity to manage the VP in the scenario individually, while multiple independent instances of the same scenario took place in the virtual world? To avoid excessive workload in the longer term, these scenarios would need to be cloned, and yet remain independent in operation.

In constructing this scenario for the T-Year students, great consideration was given to future proof the scenario, including the inevitable technological progression for more realistic 3D environments. We overcame the technical challenge to provide each PBL group with their own separate area on the virtual island to play the scenario, as well as ensuring a staff member had the ability to monitor multiple groups and restrict students from moving between groups. A major intention of this study was to illuminate these multiple group challenges and solutions for more advanced 3D worlds in future.

## Materials and Methods

### Selection of the PBL Scenario for Adaptation to Second Life

A single PBL case covering chronic renal failure was selected from a 5-week module which covers the gastrointestinal system, liver and kidneys. This module is delivered as part of the Transitional (T) year of the St. George's PBL medical curriculum where students are introduced to clinical placement for half the year and teaching within campus for the other. This whole cohort was separated into two separate groups of 130 students, one group taking this module in May and the other in July, each containing 18 PBL groups of 7–8 students, each PBL group accompanied with a facilitator in each PBL group (18 facilitators). PBL being a student-centered, collaborative and inquiry based learning activity, based on constructivism. In addition to the pedagogies of conventional PBL, our existing online interactive PBL (D-PBL) also offers the student group some degree of choice of direction of the narrative, as the scenario unfolds.

This case was used to teach the basic functions of the kidney, its role in homeostasis, complications of renal failure, transplantation, and public health issues relevant to chronic renal failure. The case was selected because (i) it included more than one location to assist in exploring the value of interactive 3D dimensional representation, (ii) it would have a reasonably high interaction with “tools” e.g., stethoscope, ultrasound machine, and computer health records.

### Second Life and St. George's Island

Warburton (18) has suggested that the immersive nature of the virtual world can provide a compelling educational experience, particularly in relation to simulation and role-playing activities. The SL scenarios and SL environment were modeled to follow as closely as possible all the pedagogical approaches and to complete the same narrative, as that covered in the D-PBL case. The additional pedagogical additions were attempting to simulate greater realism and a more immersive environment.

Second Life (SL) is a virtual world which offers users an impressionistic visual representation of a virtual world built by users or “residents.” A range of easy-to-use construction tools and a scripting language are provided by the publicly available software for the creation and editing of content in an environment that provides users with a wide range of interactions. Low costs and accessibility (via desktop/laptop device with no additional equipment required) had encouraged educators to explore the potential benefits of virtual worlds for learning. St George's had previously established an engaging and realistic environment on its island, including an orientation and training area, and a simulation of the local environment with street scenes etc. to give context to the teaching of paramedics in accidents and incidents which is key for their practice. All the environments for the scenarios and training were designed specifically for their purpose. SL offers text-chat and voice communication tools, and this enhanced the interaction with the scenario for the students in the paramedic sessions. For the purposes of this project, St George's expanded their island with the purchase of another island attached to the first, to allow more space to build the necessary multiple simulations for the different baserooms.

### Creation of the Clinical Environment in Second Life

**Figure 1:**
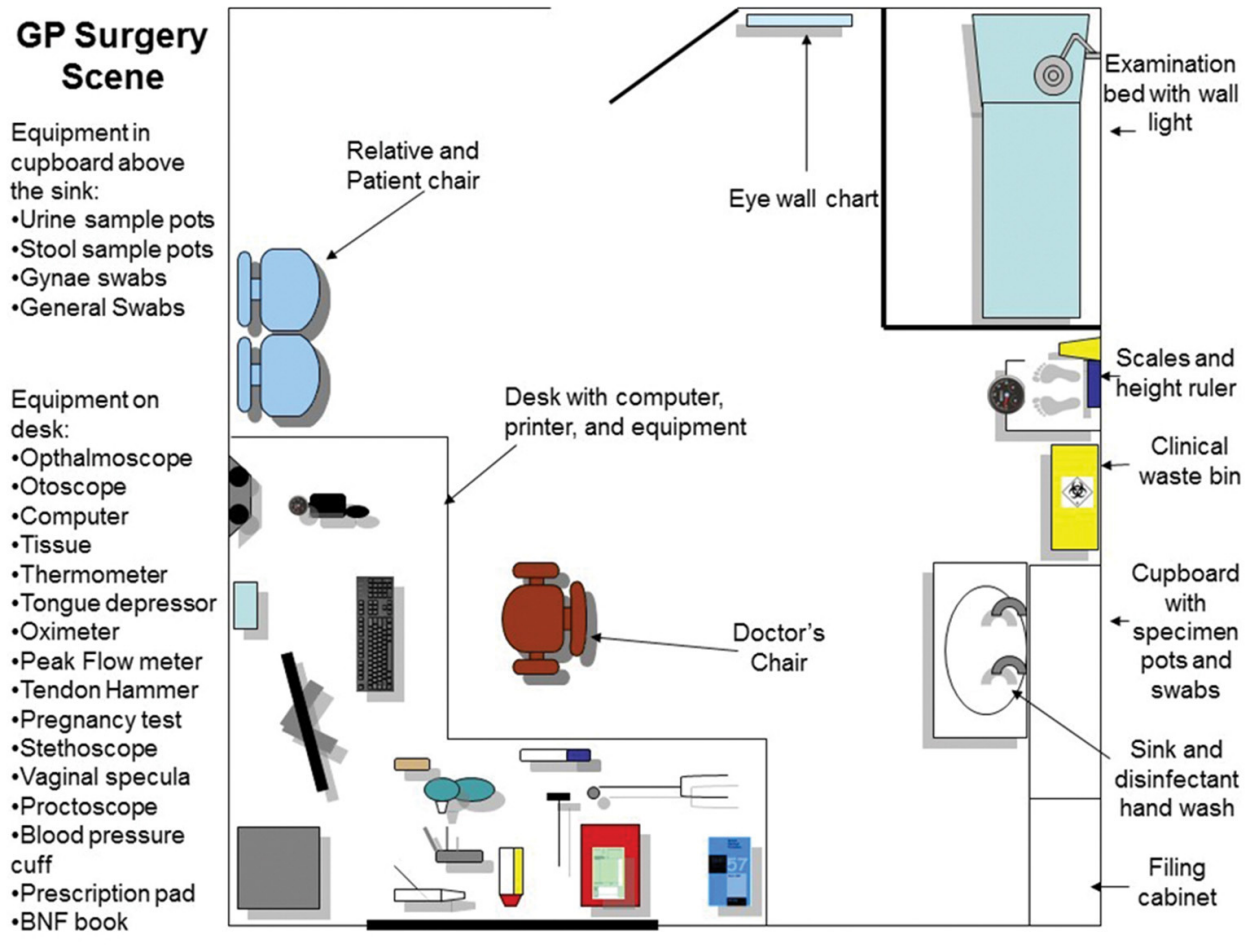
The plan of the GP surgery room. This map was used both as a design for the 3D world, and as a guide to inform students and facilitator which equipment can be found within the scenario, and its location.

**Figure 2:**
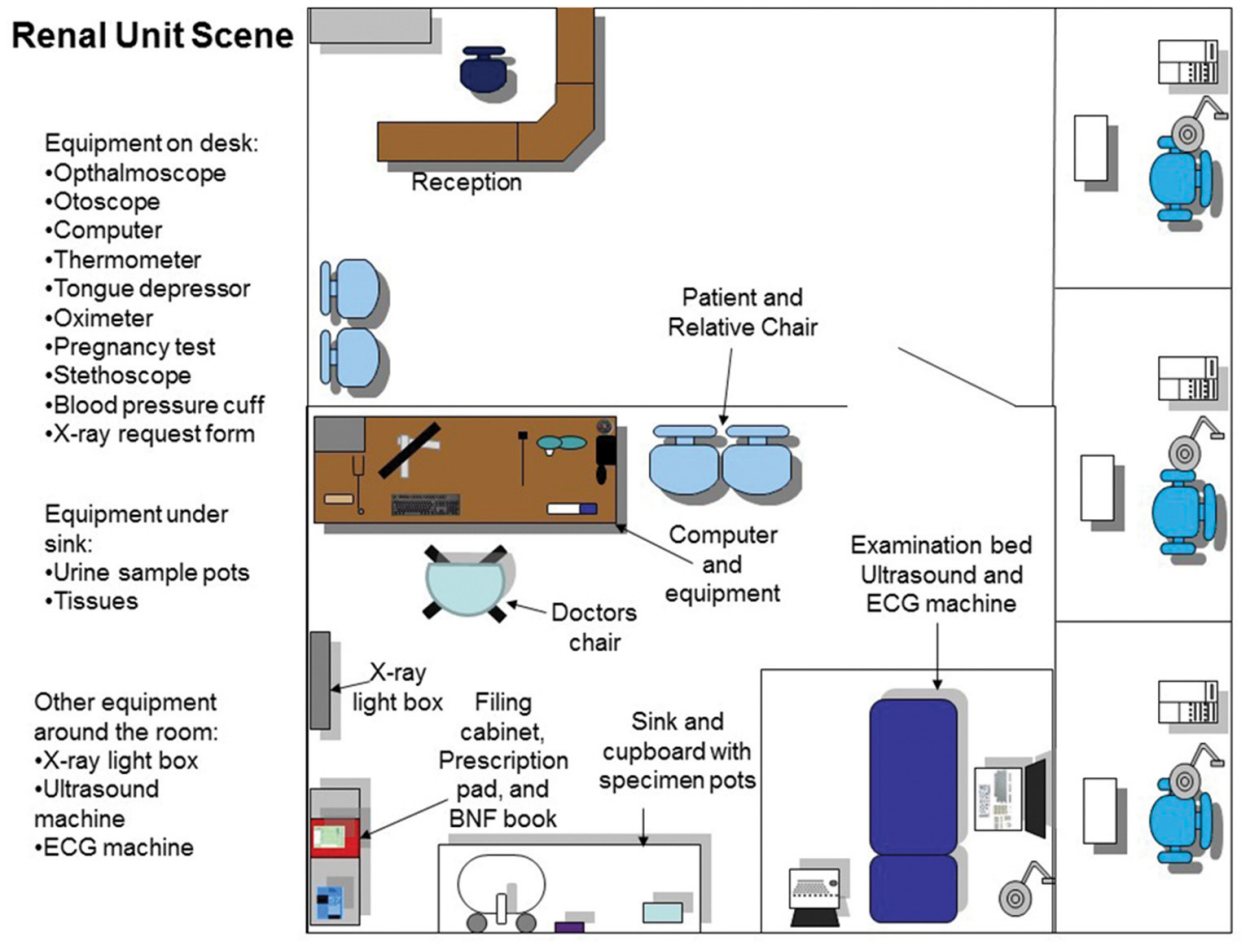
The plan and layout of the renal unit ward where the second part of the tutorial takes place. All interactive equipment is labeled and shown on the map which is provided to the students and facilitator for the session.

**Figure 3:**
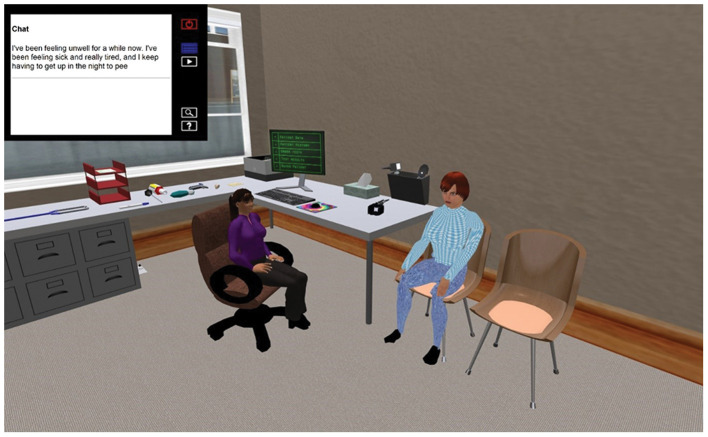
The GP surgery, showing the HUD and chat information displayed.

**Figure 4:**
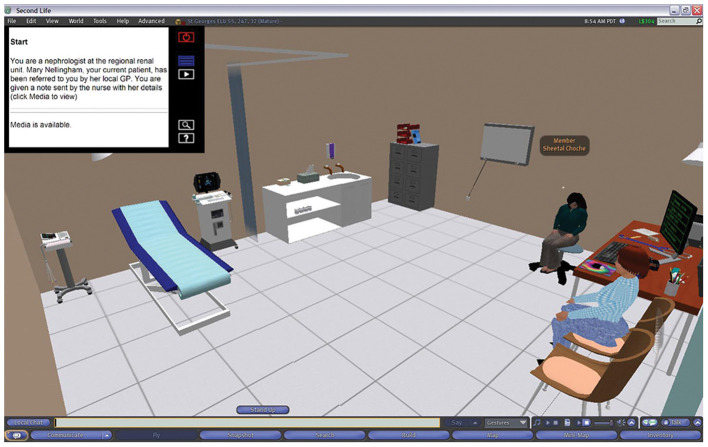
Once the case was launched, the 3-dimensional room, in this case the Renal Unit, would appear around the avatar.

The first part of the PBL case was to take place in the General Practitioner (GP) surgery (first scene), where a number of tests were required before referring the patient onwards to the renal unit within a hospital setting (second scene in the tutorial). These outline plans were created first as 2-dimensional images (Figures 1, 2), reviewed by a medical professional, who also approved the two environments (scenes) constructed within the SL world (Figures 3, 4). Complexity of the equipment within the environments was optimized to ensure the environment loaded with acceptable speed.

We did not attempt to explore or position the tutorial as a virtual reality (VR) experience for several reasons. Chiefly, we were striving to use the virtual world to foster authenticity in the decision-making process, rather than an experiential authenticity in which students might believe they are present in the environment, and to maintain the collaboration as being face-to-face in the physical room. Authenticity in decision-making is aligned with the principles outlined by Shaffer and Resnick that activities should be aligned with those in real-life practice (23). Additionally, at the time the technology was not sufficiently advanced to provide an adequately realistic environment to make VR a suitably immersive tool for students, and nor was technology such as headsets available or affordable to us.

### Development of Multiple Baserooms in Second Life

To allow 18 PBL groups to use the scenarios concurrently, 18 isolated environments were created on the island. Each environment encompassed the GP surgery and then the renal unit.

To achieve this, we used a “holodeck” tool that allowed us to build both scenarios once only but replicate this in multiple locations. The holodeck would load the next part of the scenario in response to a pre-scripted trigger that would only appear once students had completed necessary actions within the GP practice.

**Figure 5:**
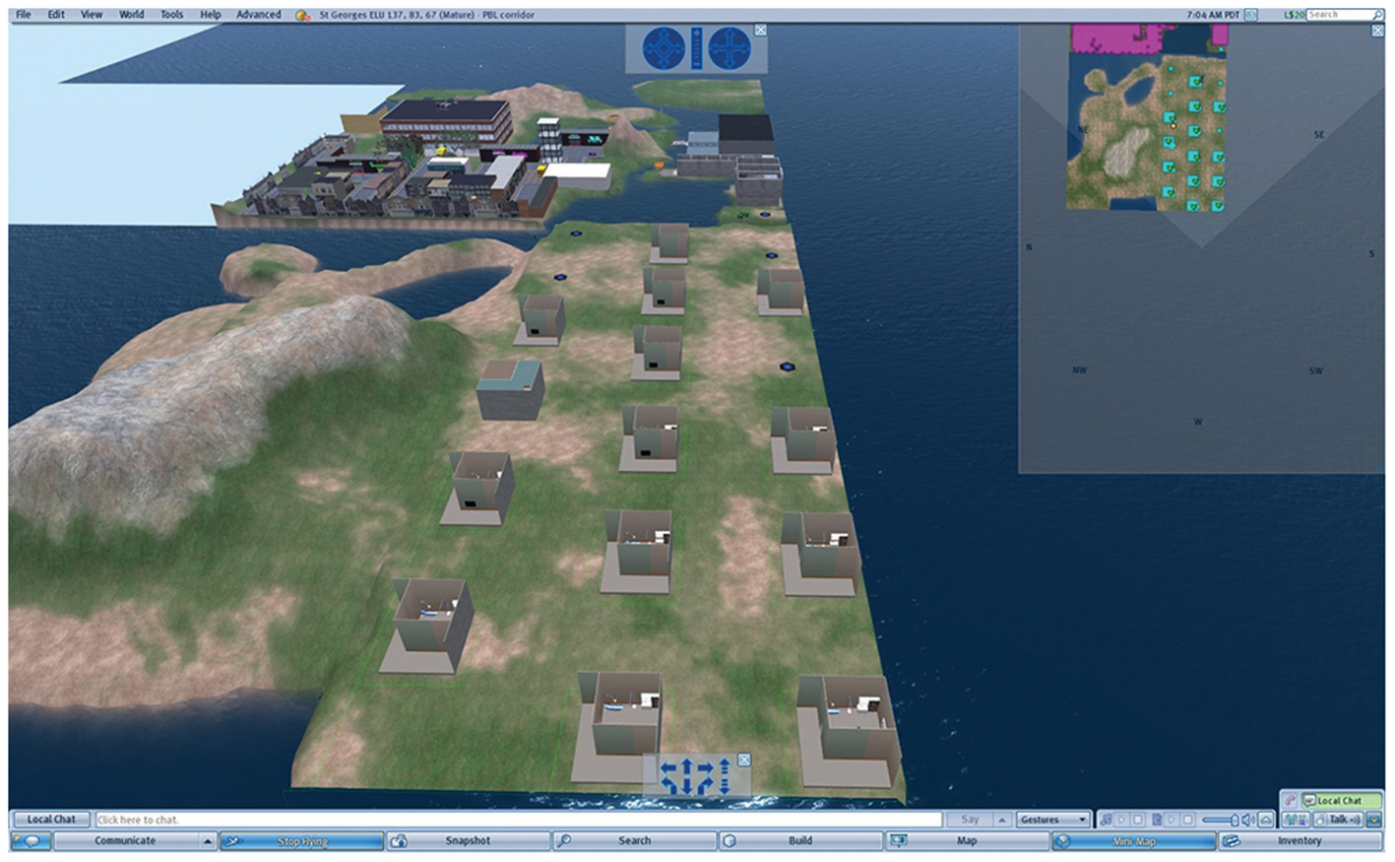
Image from within Second Life showing the virtual base-rooms on the St George’s Island with surrounding buildings and landscape. In the foreground on the right, the 18 virtual baserooms are shown by either (i) blue circles, which means the PBL group has not yet activated its virtual GP surgery, (ii) the rectangular boxes of the PBL surgery. Most groups have reached the first phase, the GP surgery, but the group 4 up on the left-hand row, has reached the renal clinic which has a different external size. A by-product of SL/PBL was that differential progress of the PBL groups could be followed.

**Figure 6:**
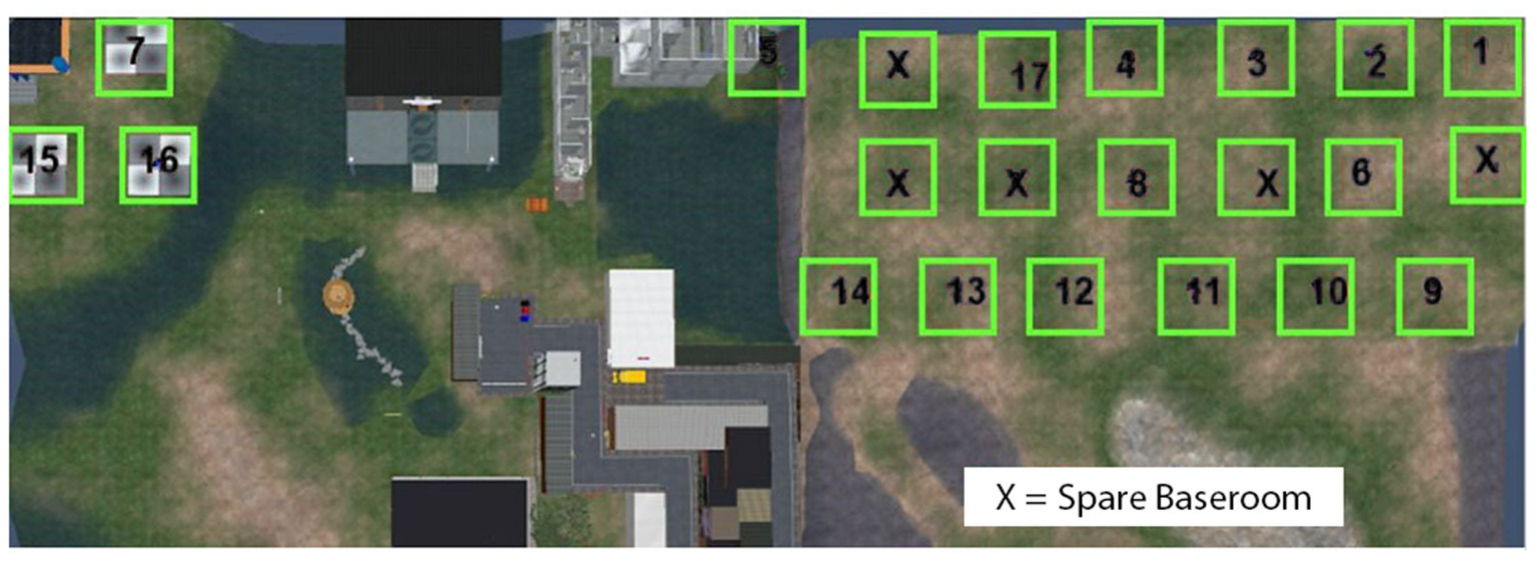
Second life Island plan of which real life baserooms will be located in which virtual baseroom. This plan was used by the development team to keep track of all rooms and to ensure there was no crossover between students.

Each PBL group was represented in the virtual world by an avatar, which one person in the group could control and use to navigate and interact with the scenario. The avatar's role was initially the doctor in the GP surgery, and subsequently as the clinician in the hospital renal unit. The holodeck which holds the scenario environment was then duplicated, to create 18 virtual “baserooms” distributed across the island (Figures 5, 6). The holodecks were carefully positioned at certain distances from each other to prevent any crossover of chat, or interaction between the baserooms.

Each PBL group was unaware of the progress of other groups due to their avatars inability to fly around the island. However, staff moderators could fly to monitor activity and help with technical issues such as progressing to the next scene within the scenario and observe the progress of each group within their baserooms. These moderators are different from the facilitators who were present in the physical room to guide the students through the scenario as they would in a normal PBL setting.

### Creation of the Virtual Patient Avatar in Second Life

The interface of the scenario was kept as simple as possible, since complicated functionality would be frustrating for students new to the environment and functionality of SL. Most of the case interactions took place using a non-player character which represented a virtual patient (VP) (from now on the non-player character will be referred to as a VP in this paper) in each scenario. This VP was physically inanimate in the scenario but provided several interfaces for learners to interact with and advance the narrative: through typed chat using “chatbot” functionality, “touchpoints” to initiate examinations, and equipment within the environment.

### Chat

The primary interaction with the chatbot was a SL text-chat interface that allowed learners to question the VP, which would respond with replies based upon the detection of keywords in the question. For example, asking a question containing the keyword “history” would result in a response corresponding to that data, so in this scenario the VP would respond with information on its medical history. The chatbot would only respond to text-chat that was prefixed with the trigger word “ask:” This prevented the chatbot from responding to other in-world communication.

The expected chat responses were assembled initially from the “paper” case, with the addition of alternative wordings identified from prior PBL experiences, before discussion with members of the Communication Skills team and an existing PBL tutor. The chat questions were believed to be acceptable in isolation, but the Communication Team found it difficult to suggest alternative triggers and questions themselves as they teach students to react to the patient depending on patient responses which includes verbal and non-verbal communication.

The mapping of keywords to text responses was stored outside the SL scenario. Standard Boolean operators (AND, OR, NOT, ELSE) provided a more sophisticated level of conversational response. This level of interactivity in the chat was appropriate for the scenario given the number of other steps the students needed to take within the given time for the session to manage the VP. A list of trigger words and keywords were provided to the PBL tutor before the session to help them to facilitate the discussions, ensuring key information from the scenario was not missed by the student group.

### Virtual Patient Examination

To carry out an examination on the VP the student groups clicked on the specific body part to interact with it. Dependent upon the body part this would trigger the physical examination information, on relevant body part e.g., limbs examination, chest sounds, abdomen examination etc. The results of the examination would be revealed in the chat and visual display (HUD) top left-hand corner (Figures 3, 4).

### Use of Equipment

Learners had access to an inventory of equipment that would routinely be used in the relevant scenario in real-world situations. Medical items such as blood pressure cuffs, cannula, stethoscopes etc. were placed around the scene within the GP practice setting, student avatars were able to touch these, and then presented with a pop-up offering a choice of possible actions. For example, by touching the octameter, the player is asked “which ear you would like to attach it to.” The object would attach itself to the VP and provide a relevant output or reading, which could change dynamically depending upon previous interactions and treatments provided. Furniture and other equipment could be used, such as computers, sinks, cupboards etc. to perform actions such as checking test results via the computer, washing hands or revealing further available equipment, like urine test strips.

### Controlling the Scenario

The scenario is driven using a screen controller that can be easily accessed through the use of the HUD, top left (Figures 3, 4), and displays media content associated with the VP, including text, images, audio, and video from an external web application created by Daden Limited (24), who were contracted as part of the previous project to develop the HUD which allowed external web content to be visible in SL from specific tiggers within the scenario. This application records the history of all actions taken by the students' avatar, and provides an interface for authoring the scenario, assembling media content and mapping keywords to text responses.

**Figure 7:**
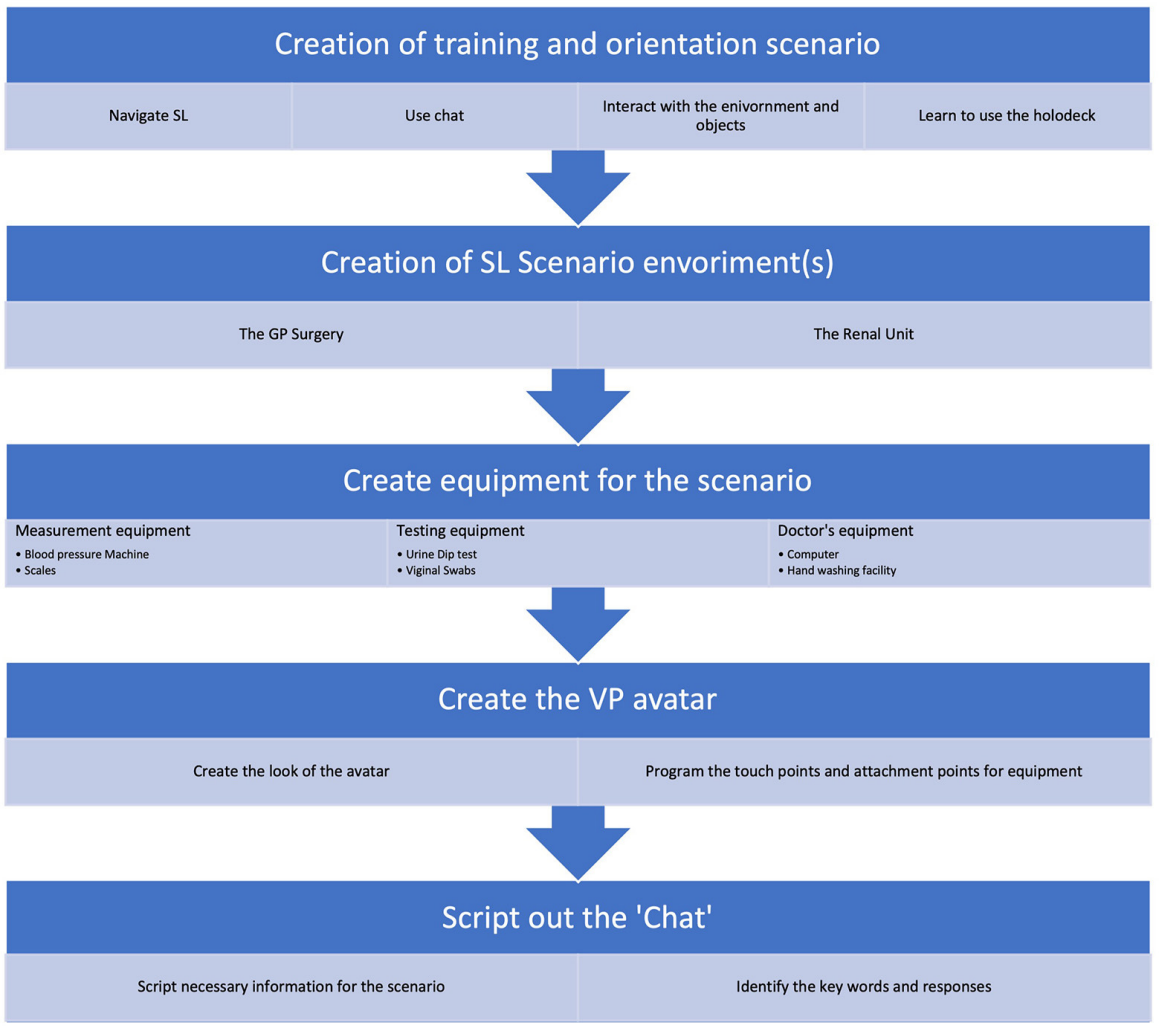
A simple flow of the order in which the elements of the scenario were developed.

By taking this scenario content out of SL into a conventional web application, we ensured that the scenario could be duplicated and used by multiple groups concurrently. Also ensuring the content was available to be used via other platforms not only within SL. The steps involved in the development of the scenario are outlined in Figure 7.

### Testing Procedure

The scenarios were evaluated at St George's during two planned testing days using volunteer students with no prior experience of SL, which accurately represented the target group. At the end of the session, they were asked to give feedback through a focus group on their experience of using the platform and completing PBL via the virtual world.

### Training of Students and Tutors in Second Life PBL

Before the PBL went “live,” two volunteer students from each baseroom were invited to a training session, in which they were taught how to navigate the St George's orientation maze using a shortened scenario similar to the PBL case. The training session ensured that two students representing each PBL group understood how to move around in SL, control their viewpoint within the scenario, use the equipment and HUD, chat with the VP, and understood how to move onto the next scene of the scenario.

Facilitators were given a demonstration of the SL scenario and provided with tutor notes during this training session to understand how the guides in the notes would help them during the session. A meeting before the PBL session was also organized to ensure all facilitators were comfortable with the SL session. Facilitators were provided with additional guides which included a map of both scenes in the scenario, the trigger words for questions to use during the chat, the list of equipment to interact with and instructions on how to use SL and move on in the scenario.

### Delivery of PBL

The PBL scenario was structured in a similar manner to traditional PBL (25), with the important distinction that the scenario now took place within a 3D environment rather than as text, in this one-off session. The students in each group, along with their facilitator were all co-located in the same physical space. The role of the facilitator within each PBL group during the SL scenarios remained the same, to guide student discussion where required. The facilitator was given a SL case-specific tutor guide, similar to the tutor guide of a conventional D-PBL case, which informed the facilitator of both the overall direction of the case, the learning objectives the students needed to cover in the case, and additional information needed for understanding and supporting the SL delivery as mentioned above.

**Figure 8:**
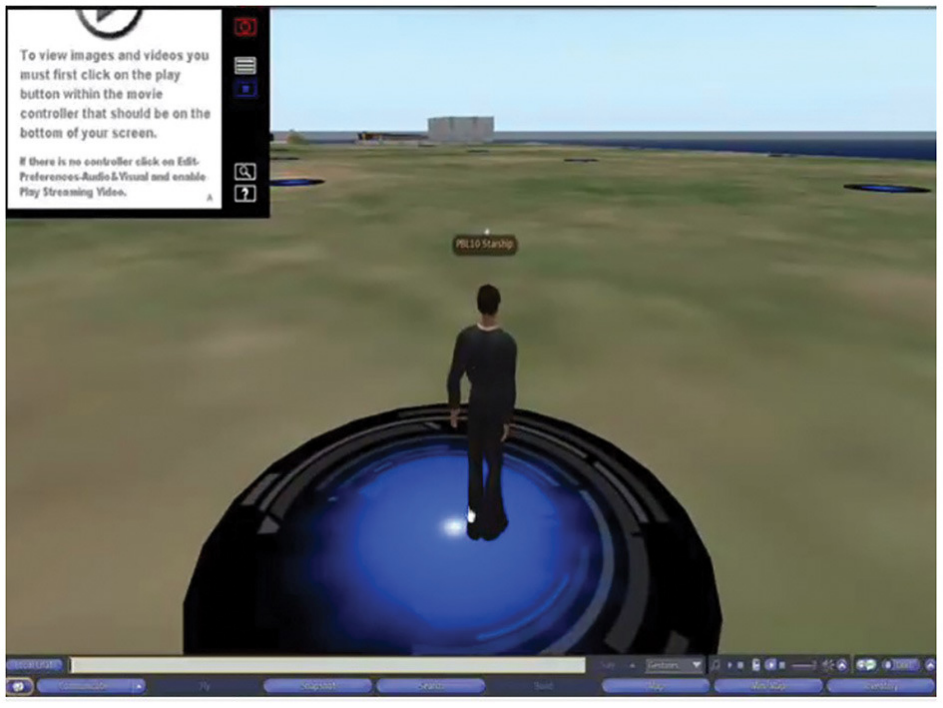
Each PBL group was set up with their own unique avatar. Either of the two students trained to use SL would log in, and their avatar would appear on their assigned holodeck, projected onto the whiteboard. Once the group was ready, they launched the case.

For the first tutorial each base room was set up with their own unique avatar log in details. The representative students in each group who had been trained for this task, logged into SL and the case was projected onto the smart whiteboard for all the students in the group. The baseroom of each group then appeared as a separate identity on the island (Figure 8), on their assigned holodeck pad (as seen as blue circles on the island).

When the group had completed their tasks in the first location (GP surgery scene), they moved to the next scene. If the group failed to complete any of the required steps, they would be prompted to carry out further investigations before moving on. The second location (scene) of the scenario was set in the hospital renal unit. As before, the group discussed how they should proceed, and which investigations should be carried out within the second location.

The session lasted 3 h, the same as the D-PBL session, and the tutorial adhered as closely as possible to the traditional process for either a paper case or a D-PBL case. The students generated learning objectives whilst exploring the scenario, and “interacting with the VP.” After the tutorial, students carried out their self-directed learning as usual from the learning objectives generated, for reporting back and discussion at the next tutorial.

Only the first tutorial was delivered in SL, the same students went on to complete the second tutorial which was delivered as St George's standard interactive D- PBL case for the normal allocated 3 h, but this second tutorial was not evaluated as part of this study.

### Data Collection and Analysis

Data collection was principally captured in the form of structured student and tutor surveys and a student focus group after delivery of the SL PBL session. The survey was created with the aim of evaluating the virtual worlds resource and had not been validated.

Descriptive statistics were calculated for survey items. Focus group comments and open-ended survey responses were analyzed by an external member of the project team using a directed content analysis approach (26) and the tool Atlas.ti (26), to provide further context and background to the themes identified from the survey.

## Results

### Student Survey

#### Experience and Perception of Virtual Worlds Prior to the Intervention

Two hundred forty-four students out of a possible 260 completed the survey, representing 34 out of the possible 36 PBL groups. As shown in Table 1, 177 (73%) students had not used virtual worlds before. Fifty-two (21%) had used them for entertainment purposes, though in providing further details many qualified this by indicating that their experience was in computer games such as the Sims (27) which are very different in style, scope and user experience to SL. 10 (4%) students had previous experience of using virtual worlds for education purposes.

**Table 1:** Previous experience with virtual world.

**Answer options**	**response** **Percent (*n* = 244)**	**Response** **count**
No	73%	177
Yes, for fun	21%	52
Yes, for education	4%	10
Yes, for another purpose	2%	5

One hundred seventy-seven students had not previously used virtual worlds and were asked to provide details from a multiple-choice list. One hundred four students cited a lack of interest as their reason for never having used them, with 82 pointing to not having a prior need to do so. Thirty-seven students saw no value in virtual worlds, while 20 students were unaware of their existence. Twelve students identified that they did not have access to a computer that can support virtual worlds, while 9 were not comfortable with using the software.

Nevertheless, despite this general lack of experience in virtual worlds 152 (62%) of the students thought that virtual worlds could provide useful learning resources for medical education, with 88 (36%) answering that it would not be useful (4 students did not respond). One hundred thirty (53%) students reported that they did not require any more guidance than was provided to participate in the PBL, with 25 (10%) indicating that more guidance was needed, and 89 (36%) electing not to answer the question. The students were also asked “*if the time taken to learn how to use the platform was off-putting*” and the response was mixed, with 131 answering “yes” (54%), 109 (45%) answering “no,” and 4 students skipping the question.

#### Assessment of the Intervention

##### Quantitative Analysis

The students were asked to rate a range of Likert items using a 4-point scale, based upon “how easy was it to use the [SL] features” (Table 2). Mean responses were calculated by assigning a numeric scale of 1.

**Table 2:** Ease of use of the features during the use of virtual world.

		**Response count**				
**Answer options**	***n***	**Very difficult**	**Fairly difficult**	**Fairly easy**	**Very easy**	**Mean rating**
History taking	239	36	100	92	11	2.33
Using the medical equipment	241	12	31	149	49	2.98
Conducting a physical assessment	237	13	57	125	42	2.83
Using the HUD (Heads-Up Display Screen)	229	18	63	125	23	2.67
Viewing media e.g., images	238	17	54	138	29	2.75
Moving between the GP surgery/renal unit	233	24	52	126	31	2.70
The scenario as a whole	235	33	71	120	11	2.46

*241 students answered this question (3 students skipped)*.

The easiest feature was using the medical equipment with a mean rating for ease of use of 2.98 followed by conducting a physical assessment with a mean rating of 2.83. By contrast, the most difficult features to use were: (i) history-taking (2.33) and (ii) the scenario as whole (2.46). One hundred forty-five of the students reported that they received the expected responses from the virtual patient chatbot most of the time, although 84 felt that they rarely received the expected response, and 15 skipped the question. One hundred fifty-four respondents felt that the scenario was not realistic, compared with 85 that did. One hundred thirty-five respondents stated that the information in the scenario was presented clearly, while 104 respondents felt that the information presented was unclear.

##### Qualitative Analysis

The survey included some open-ended questions (Table 3) which provided further insight into what the students expected from the scenario and yielded some key themes. Approximately half of the students (78) who responded considered that SL promoted decision making, having to think about different options rather than choosing from a given list. For example, “*allowed us to also think outside the box rather than to just be given the scenario.”, “.Think of ways to investigate patient rather than pick from list.”*

**Table 3:** Categorization of responses to two open-ended questions (“what worked well during SL PBL,” from 155 students) and (“what didn’t work well during SL PBL”; from 199 students).

**Worked well**	**Didn't work well**
1. Enhanced decision making (78) 2. Enjoyable/interesting learning experience (40) 3. Design of the scenario during the “examination” (36) 4. Possibility of interacting with the patient during history taking (30) 5. Capacity of enhancing teamwork and discussion (27) 6. Realistic (22) 7. Interactivity (6) 8. Feedback (5)	1. Slow/time consuming (103) 2. Question/answer process during the history taking (86) 3. Technical design: glitches and appearance of the scenario (58) 4. Sequence of facts was confusing (45) 5. LOBs not achieved (20) 6. Team work was hindered (19) 7. Not realistic (19) 8. Lack of some necessary examinations (5)

In contrast, although the ability to examine the patient was praised to some extent, this was an area that the students found limiting. Learners reported being aware that they were not having a conversation with the patient, but that the patient was simply responding to a pattern-matched phrase or word. With this knowledge they then altered their behavior to use minimal phrases and words, one student stating “*you could easily pick up that you're just recognizing key words, and you just put in keywords.”* Student opinion on history-taking was mixed; though some found it engaging, the majority reported that the process question/answer during the history taking did not work well; “*the patient didn't understand even simple questions during the history taking process”* and that “*the responses from the patient were not appropriate.”* Responses to the examination elements were more positive and the “key elements” (tests and equipment) were present, realistic and easy to use.

One area in which opinions amongst the students differed greatly was around the realism of the scenario. Some students praised the realism of the approach to addressing the patient: “*I think it is realistic in a way, because clinically, the processes are there that you have to go through systematically; history, examination, investigation, and referral.”* However, other students expressed frustration with the boundaries of the realism, commenting that “*if you're a first year and you've never been into a hospital before, it's quite fun to see what a room might look like, but you know, there's no patients, and no people. There's no kids running around. It's not realistic.”* Nevertheless, some students considered that the SL PBL was “fun” and “interesting” and encouraged discussion.

Students disliked the lack of guidance and ordering of events, which is a feature of virtual worlds and found the SL platform difficult to use, especially controlling the VP and moving from one scenario to the other.

#### Preferences on the PBL Teaching Methodology

The students were asked to rank the different forms of PBL: paper PBL, online D-PBL and virtual PBL. One hundred eighty-five (77.08%) of the 240 respondents ranked D-PBL in 1st place; 36 ranked paper PBL in 1st, and only 19 ranked virtual worlds 1st. In all rankings D-PBL was the favored methodology, SL was consistently lowest.

The students were almost equally divided between those who believed “*there was value/potential for value in this method of PBL*” (51.1%), and those did not. However, 77.2% of the students reported that SL “*PBL was hindered by the style of scenario*.” A smaller percentage (53.3%) though it “*hindered effective collaboration,”* and 13% thought it “*aided collaboration.”* Only 3% of students thought that the “*PBL was improved by this style of scenario*.” Less than half (47.4%) of students believed that they covered the appropriate Learning Objectives' while using the SL PBL.

### Facilitator Survey

Sixteen out of the 18 facilitators completed the short facilitator survey, mainly composed of open-ended questions exploring the effectiveness of SL PBL and response of the students.

Most facilitators considered SL PBL is less effective than both the interactive D-PBL and the paper PBL. Some commented that it was more difficult to identify learning objectives compared to the other methods. Only 25% reported that the SL PBL was effective. In general, facilitators agreed with student preferences for the different forms of PBL, with D- PBL first (60%), but SL was second with 27%, and paper last.

Most facilitators (60%) reported that it was more difficult to facilitate the virtual world PBL sessions, though a proportion believed that increased training and practice would improve the facilitation process. Furthermore, ~30% of the facilitators believed that the SL PBL could be useful if further developed.

Facilitators believed that during the facilitation of the SL PBL, the students were actively engaged but at the same time the dynamic was more complicated than usual and 80% of the facilitators believed that technology, at least occasionally, “got in the way of the learning.”

Some facilitators found SL case easier tutoring the second time around, with one facilitator stating that it “*was smoother but I think that was due to the fact I have already done this case before it was easier to make use of the virtual map to find appropriate equipment for examination and investigations.”*

## Discussion

This study investigated the feasibility of running multiple baserooms simultaneously, but also independently, each with its own group-controlled avatar. We had expected that the virtual world may be ultimately lacking in realism, but nevertheless would provide a pointer to the feasibility of creating separate simulations for each PBL group. This would then address an overarching objective of the PBL curriculum, to provide a unique experience for each group, with the students in charge of the management.

In practice, the theoretical process for establishing the individual baserooms worked very well. The two students who had volunteered to be trained from each PBL group were capable of managing the necessary functionality of the avatar, HUD and chatbot features.

The survey results indicate that students considered that SL PBL provided a decision-making opportunity which encouraged them to think and discuss the different options. Some of the students that had previously used virtual worlds, enjoyed the experience for its entertainment value and believed it to be more realistic, despite the need for design improvements. However, whilst a minority reported that the SL experience felt more realistic, most did not, and the students clearly preferred their simpler text-based online interaction, the D-PBL experience. This finding is also in agreement with Loke's review of proposed mechanisms of learning in virtual world experiences (28) which found that, contrary to a popular conception, students do not appear to experience a physical presence of real-world phenomenon through their virtual world actions; they do not perceive virtual worlds as “learning by doing.”

A repeated common theme in student/facilitator surveys and focus groups was it would be necessary to heavily review and improve the design of this tool to make it viable. It was clear that the software had not reached state-of-the-art which would allow students to feel comfortable with either technology or the level of realism. Nevertheless, it is instructive to consider whether, even if realism improved, students would gain significantly from the experience. Students made the point that the online D-PBL already contains many of the characteristics that we are seeking with the use of SL PBL, and therefore further attempts at realism were not necessarily productive or cost-effective. As technology evolves in areas of VR and AR lessons learnt from this study some of those listed in Table 3 could be applied to future studies. It is possible that the assessment tool developed by Fu et al. (29) to assess student enjoyment of games, could be adapted for future assessment of student satisfaction with 3D educational tools.

This study would appear to be at odds with other findings which have explored the value to students of greater realisms in PBL settings (22), but in these studies, students did not have a comparison with interactive scenarios in a non-3D World. Consequently, this may be the first time for 3D environments that a fair comparison has been made of one interactive tool vs. another i.e., one text-based and 2D, the other a more complex 3D world. More normally the 3D world comparison is made of one interactive tool vs. a static linear, non-decision-making tool, and thereby negates any advantage that these interactive environments may possess over the more linear learning and teaching opportunities. Since this work, there has been a number of other studies and developments in technology for use in education where (30) found increased student learning performance from the use of collaborative learning environments.

A non-controlled variable may have influenced the subjective opinion of students concerning the merit of virtual PBL. The students considered that it was not appropriate to organize this session 3 weeks before the exams, and this may have influenced their perception of virtual world PBL.

What has emerged from this study is a clear view of what students would require before a 3D world would have any advantage (in terms of student engagement) over their existing interactive, text-based system, namely: improving the technical design and organization of the content; increasing the technical sophistication of the virtual world, and reducing the time needed to understand the tool.

An attempt to improve the technical design, would encounter the issue that the open-endedness of a virtual world provides few clues to students for how to progress further in the scenario. The participants' role is not intuitive. By contrast, structured gaming environments present defined possibilities for action. Games are more intuitive, they can guide the participants to know what to do next, and when the activity is finished. Likewise, text also offers a similar sense of structure.

Improvements to a virtual world would require technologists, physicians, and educators to work together to consider all the possible options that would allow the students to cover the scenario satisfactorily, and it is questionable whether this effort would be worthwhile, with so many challenges to overcome.

An obvious example is the chat function. In terms of content design, the chat does not work well enough to be useful. Improved mapping might help, but it must be borne in mind that students and practitioners ask questions in their own style and that mapping may require extensive semantic analysis and a very large vocabulary. With advances in AI the approach to “chat” within the scenario could be explored with tools outside SL which have developed for more realistic interaction and responses. Similar challenges are presented in an open-ended environment with a number of available tests when assessing the patient; those offered are likely choices, rather than unlimited choices.

Aside from the technical limitations of the platform, there are some limitations to the study which must be considered. Primarily, the generalisability of our results are limited by the fact that participants were from a single institution and setting, and received one intervention in the virtual world. There were also a number of non-controllable factors that may have influenced our results, such as the tutorial taking place prior to an exam period which was the students' primary focus. Although participation in the tutorial was part of the regular curriculum, completion of the survey was not mandatory, and a more detailed analysis of responses per group would be needed to properly assess and identify patterns in the responses that might indicate the presence of attrition bias. Similarly, the design of our survey was not validated in this context prior to the study and our analysis does not allow us to account for intra-group correlation, which has the potential to introduce a further bias into our results. These limitations necessarily mean that more evidence would be required to support our conclusions and establish if our findings can be generalized beyond the context of this study.

One potential objective had been to consider running PBL groups for students in different geographic locations as Melus-Parazon et al. (31) had achieved in primary health care settings, but the technical difficulties removed this possibility. A summary of what worked well for this study from the students perspective and what could have been better is provided in Table 3 of the Results section. From this we can see conflicting views however it is hard to draw a conclusion from this due to students only doing one tutorial in SL.

It is increasingly recognized that many e-learning interventions may be far more costly than they need to be “*Educators often seem to use cutting edge (read that as expensive) technologies because they are available, rather than because they add value commensurate with the higher cost*” (32). Though this study points the way toward a process for running PBL sessions in 3D environments, and at the same time shows the ability to monitor those session more completely than is possible in conventional non-virtual world PBL, it is clear that it would seem to require a very considerable change in the technology before this process can be useful to both learners as well as educators.

Nevertheless, the pursuit of more engaging learning activities, including virtual realities, may well be worth the effort. Norman et al. (33) has noted that a learning tool does not need to mimic real life, it is the learning experience that needs to reflect real-life. Recent events have forced radical changes in face-to-face modalities for PBL which will carry the risk of a more remote and less engaging learning experience. Students may not have seen “the point” of virtual realities beforehand, in their face-to-face tutorial groups, but the switch to online PBL may promote a search for a more engaging experience, by students and tutors alike.

Immediately before the COVID-19 pandemic, Savin-Baden (34) noted that ideally PBL should include practices such as gaming, emotional learning, playful learning as well as student-led cooperation in mentorship, technology support, and even co-production. The issues that this study faced are similar to those found in a recent study by Sancar-Tokmak and Dogusoy (35) who explored using SL to solve the high dropout rate in a Distance Learning Center. Although the learners could recognize the opportunities that SL could provide for PBL, they preferred alternative learning methodologies because of access and usability issues.

It is to be hoped that in accepting the pedagogic losses we experience from the reduction in face-to-face teaching, we can provide compensations in other directions, online. Perhaps cooperative online learning that includes virtual realities may help to contribute, as Castelo-Branco et al. (25) puts it, “a pedagogy of imagination and surprise.”

## Data Availability Statement

An anonymised version of the data supporting the conclusions of this article will be made available by the authors upon direct request.

## Ethics Statement

Ethical review and approval was not required for the study on human participants in accordance with the local legislation and institutional requirements. Written informed consent for participation was not required for this study in accordance with the national legislation and the institutional requirements.

## Author Contributions

All authors listed have made a substantial, direct and intellectual contribution to the work, and approved it for publication.

## Conflict of Interest

The authors declare that the research was conducted in the absence of any commercial or financial relationships that could be construed as a potential conflict of interest.
